# Using Eye Movement to Control a Computer: A Design for a Lightweight Electro-Oculogram Electrode Array and Computer Interface

**DOI:** 10.1371/journal.pone.0067099

**Published:** 2013-07-03

**Authors:** Eduardo Iáñez, Jose M. Azorin, Carlos Perez-Vidal

**Affiliations:** Biomedical Neuroengineering Group, Miguel Hernandez de Elche University, Elche, Spain; Glasgow University, United Kingdom

## Abstract

This paper describes a human-computer interface based on electro-oculography (EOG) that allows interaction with a computer using eye movement. The EOG registers the movement of the eye by measuring, through electrodes, the difference of potential between the cornea and the retina. A new pair of EOG glasses have been designed to improve the user's comfort and to remove the manual procedure of placing the EOG electrodes around the user's eye. The interface, which includes the EOG electrodes, uses a new processing algorithm that is able to detect the gaze direction and the blink of the eyes from the EOG signals. The system reliably enabled subjects to control the movement of a dot on a video screen.

## Introduction

People suffering from neurological conditions leading to severe motor disorders are not able to use classical communication devices to interact with computers, such as a keyboard or a mouse. In these cases, computer commands must be generated without using arms or hands, e.g. using voice recognition [1], [2], brain-computer interfaces (BCI) [3]–[6] or eye movements [7], [8].

In this paper, an interface that allows people to interact with computers using their eye movements is presented. In order to detect eye movements, the system uses electro-oculography (EOG). EOG detects the eyes movement by measuring, through electrodes, the difference of potential between the cornea and the retina [9]. EOG has been used in previous works to interact with different devices. For instance, our research group used EOG to control a robot arm [7], [10]. In [9], [11], EOG was used to guide a wheelchair, and in [12], an EOG interface was used as a first person navigation system.

However, few or none research groups are developing a whole system that includes physical support of electrodes, electronics and the communications with the computer. A new device for holding dry EOG electrodes is designed in this work, making a step forward in the way to obtain a commercial pair of EOG glasses. They include the dry EOG electrodes, the electronic circuitry to acquire and send the EOG signal/s and the batteries. This system could be used (put on and removed) easily and very fast (avoiding the use of plasters to manually fix the electrodes). Furthermore, it could allow the popularisation of the EOG technology to interact with devices. The EOG pair of glasses have been manufactured using a 3D printer to obtain a prototype.

The interface presented in this paper uses a new processing algorithm to detect eye movement that improves the characteristics of a previous work [10]. The new algorithm is able to detect the gaze direction of the eyes (right, left, up and down) as well as the users' blink. This algorithm is more robust and efficient than the previous one. Furthermore, it reduces the time required to obtain the gaze direction. The algorithm works using time windows of 1, 0.5 or 0.25 seconds. The algorithm is even able to detect eye movement performed between two processing algorithm windows. The pair of glasses and the new processing algorithm have been tested intensively by several volunteers. The results of the experiments have shown that the whole system has high reliability.

## Materials and Methods

### Electro-oculography

**Figure 1:**
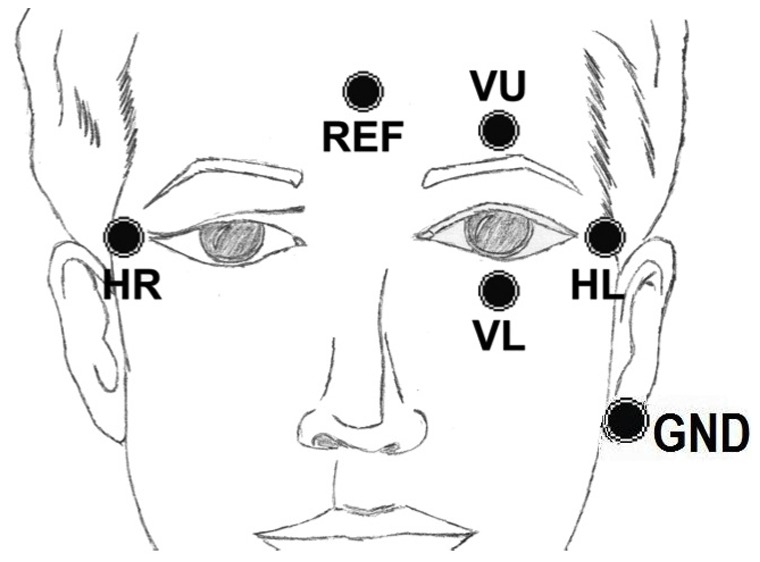
Electrode location. Shows the position of five dry flat electrodes placed on the face around the eyes to detect vertical and horizontal movements.

Electro-oculography (EOG) is a usual method for registering eye movements [13]. It is based on the fact that the eye acts as an electrical dipole between the positive potential of the cornea and the negative potential of the retina. In normal conditions, the retina has a bio-electrical negative potential related to the cornea. Thus, rotations of the ocular globe cause changes in the direction of the vector corresponding to this electrical dipole. The recording of these changes requires placing five dry flat electrodes on the face around the eyes, as can be seen in Figure 1. This figure shows the position and names of each electrode used (VU, VL, HR, HL and REF). Two electrodes are placed on the right and the left of the eyes (HR and HL) to detect horizontal eye movement. Vertical movements of the eyes are detected by two electrodes placed on the top and bottom parts of the eye (VU and VL). The reference electrode (REF) is placed on the forehead, approximately in the FPz position of the International System 10/20 [14]. Finally, the ground (GND) is placed on the ear lobe.

The EOG signal varies from 50 to 3500 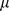
V, with a frequency range of about DC-100 Hz between the cornea and the Bruch membrane located at the rear of the eye [9]. Furthermore, its behaviour is almost linear for gaze angles of 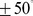 horizontal and 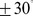 vertical [15].

### EOG Amplifier

As amplification and digitisation device, the g.USBamp amplifier from g.tec, with a frequency sample of 120 Hz, has been used. The g.tec device provides some internal filters. A bandpass filter between 0 and 100 Hz, and a Notch filter of 50 Hz to eliminate the network noise have been applied. The software used to register and process the EOG signals has been developed in Matlab using the API (Application Programming Interface) provided by the device (g.USBamp MATLAB API).

Electrodes from Easycap (model E273) have been used to register the EOG signals. These electrodes are flat Ag/AgCl electrodes of 12 mm diameter with a light-duty cable and 1.5 mm-touchproof safety sockets. The advantage of this type of electrode is that they do not require conductive gel to operate, so there is only a need for some cleaning abrasive gel on the skin before putting the electrodes on, which makes the placing of the electrodes on the user, easier and faster.

### Pair of EOG Glasses

A pair of glasses have been designed to integrate all electronic (EOG electrodes, printed circuit board, batteries and communication module) and mechanical elements (covers, electrode holders, lenses and frame), trying to obtain the most aesthetic device as possible and arriving further than to the functional design carried out by other similar works [16], [17].

#### Design

**Figure 2:**
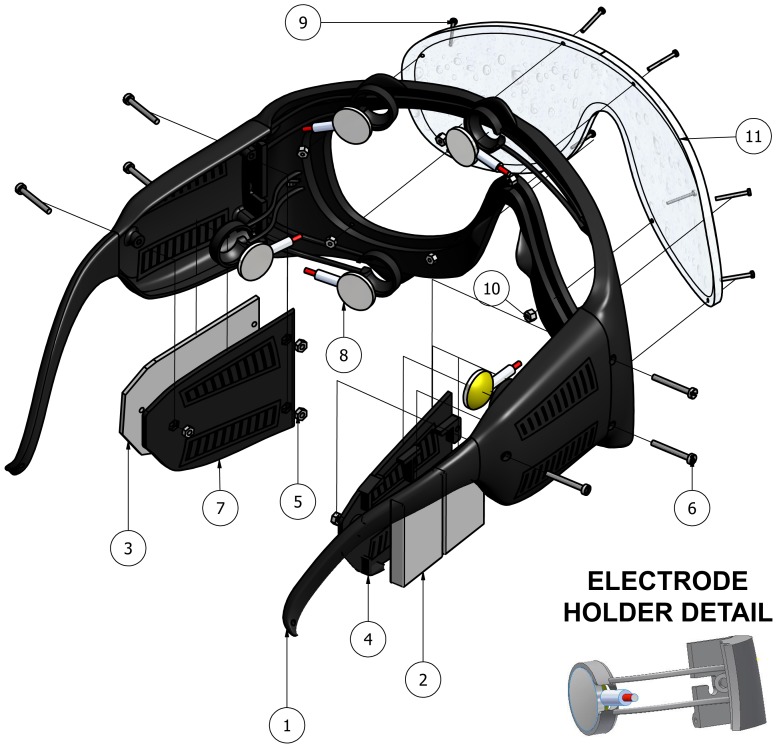
Pair of EOG glasses. Autonomous device designed to hold five dry flat electrodes, electronics (including signal preprocessing and wireless communication) and batteries. The electrode holders can be seen in bottom right corner of figure. Dry flat electrodes are held by flexible arms (electrode holders) to adapt the sensor to the user skin. Material and geometrics have been considered to generate a force of 0.2 N.

After an iterative process of design and checking, the device shown in Figure 2 has been obtained. The list and description of elements labelled in this figure can be find below:

1. Hole/s. At the end of each leg there is a hole to insert an elastic band so that the pair of glasses fit tightly to the head in order to avoid their falling off due to sudden movement or shaking.
2. Two batteries model 616-0212 (Li-Polymer, 3.7 V/250 mAh). Size: 5×20×30 mm (the same batteries used in iPod Shuffle 1st Gen).
3. Printed circuit board (PCB) with pre-processing components and communication module to transmit the signal to a computer.
4. Cover for the batteries (right side).
5. Nuts (to fix covers) placed inside the glasses.
6. Screws (diameter 1.6 mm, L 14 mm) to fix lateral covers.
7. Cover for the PCB (left side).
8. Dry flat electrodes (model E273 from Easycap).
9. Screws (diameter 1 mm, L 10 mm) to fix front lens.
10. Nuts (to fix the front lens) placed inside the device.
11. Front lens (molded plexiglass).

The five dry flat electrodes to register the EOG signals (VU, VL, HR, HL and REF) are held by a group of electrode holders. In bottom right corner of Figure 2, an electrode holder detail is shown. These holders are designed to obtain a suitable contact with the skin.

#### Finite element analysis

The objective of this step involves designing each electrode holder and validating the shape and thickness in a Stress Analysis environment. Stress Analysis (of Autodesk Inventor software) is used to determine the stress, displacement and safety factor of the design. The work flow will be repeated until the shape and thickness of the rod is optimised against the design criteria.

The following design criteria have been chosen based on researchers' experience:

YIELD STRENGTH (MPa): 276, DEFLECTION (mm): 1.25, SAFETY FACTOR: 2.0 (values applied only to the electrode holders design).

The key terms of the design process have been based on [18] and can be described as:

- Assembly.

Two or more components (parts or sub-assemblies) are considered as a single model. An assembly typically includes multiple components positioned absolutely and relatively (as required) with constraints that define both size and position. Assembly components may include features defined in place in the assembly. Mass and material properties may be inherited from individual part files.

- Stress analysis.

A previous analysis to check that the model is stable statically and dynamically and free from divergence on application of external loads and frequencies is required. In this work, stress analysis is used to ensure that the material and geometry of electrode holders can handle the loads without breaking or failing.

- Simulation.

The analysis of the each electrode holder uses Stress Analysis to optimise the shape and thickness of these elements. Stress Analysis is used to analyse the material at the point of maximum load on the lever.

- Von-Mises Stress.

Three-dimensional stresses and strains build up in many directions. A common way to express these multidirectional stresses is to summarise them into an Equivalent stress, also known as the von-Mises stress [19]. A three-dimensional solid has six stress components. Sometimes a uniaxial stress test finds material properties experimentally. In that case, the combination of the six stress components to a single equivalent stress relates the real stress.

- Displacement.

**Figure 3:**
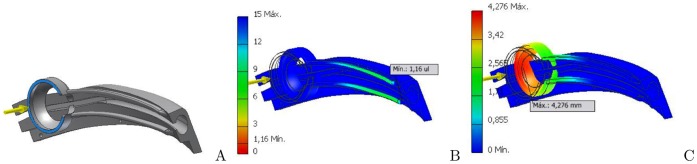
Finite elements analysis. A) represents the force vector applied to the electrode holder. B) shows the strain in the base of the electrode holder. C) shows the strain in the electrode holder ring.

Displacement is the amount of stretching that an object undergoes due to the loading. For this work, the displacement is limited by the structure of the glasses as can be seen in Figure 3.

- Safety factor.

All objects have a stress limit depending on the material used, which is presented as material yield or ultimate strengths. If VisiJet SR200 has a yield limit of 26.2 MPa, any stresses above this limit result in some form of damage.

The safety factor is how much stronger the system needs to be for a given load. The factor of safety can be calculated as the ratio of the maximum allowable stress (Yield Strength) to the equivalent stress (von- Mises) under the maximum load.

Figure 3 shows the electrode holder in image A, while images B and C show the areas subjected to stress. A safety factor of around 7 can be seen in image B, and a maximum deformation of 4.276 mm can be seen in the image C of this figure.

#### Prototype printing

**Figure 4:**
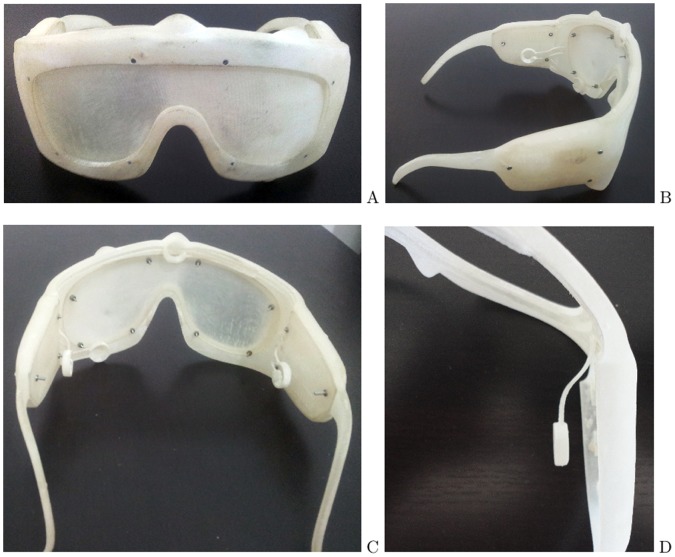
Prototype printing. A) shows the front side of the device. B) shows the left leg of the glasses with the PCB cover and several electrode holders. C) shows the back side of the glasses, where the M1.6 screws are uncut. D) glasses right side showing a detail of one electrode holder.

This prototype has been manufactured using a well known rapid prototyping technique, 3D printing. Rapid prototyping refers to a broad category of processes used to build models layer by layer from computer-generated STL data. Two common forms of rapid prototyping are stereolithography (SLA) and selective laser sintering (SLS). After the design is obtained (and shown in Figure 2), the STL file is generated and sent to a 3D Systems HD3000 printer, that after 12 hours generates the pair of glasses shown in Figure 4. The printer was configured in HD mode, that means a resolution of 328×328×606 DPI (xyz).

The duration of the printing was 20 hours and 19 minutes. The cost of printing material (in HD mode) was 20.34 euros of resin and 54.72 euros of supporting material. Another option could be the printing in UHD mode. The printing should be performed in two steps (due to the printing area reduction in UHD mode) and would take 69 hours. The cost in this case is slightly higher due to the position in which the glasses must be placed. The cost of printing material (in UHD mode) would be 23.95 euros of resin and 59.44 euros of support material.

### EOG Processing Algorithm

To detect the eye movement from the EOG signals, a new processing algorithm, which improves the characteristics of a previous work [10], has been developed. The new algorithm is able to detect the gaze direction of the user's eyes (right, left, up and down) as well as the users' blink. This algorithm is more robust and efficient than the previous one. Furthermore, it reduces the time required to obtain the gaze direction. The algorithm works using time windows of 1, 0.5 or 0.25 seconds. The algorithm is even able to detect eye movement performed between two processing windows. The algorithm considers that the users perform a fast movement of their eyes in the desired direction, returning then to the centre position. This way, the users are always focused on the graphical user interface of the computer.

**Figure 5:**
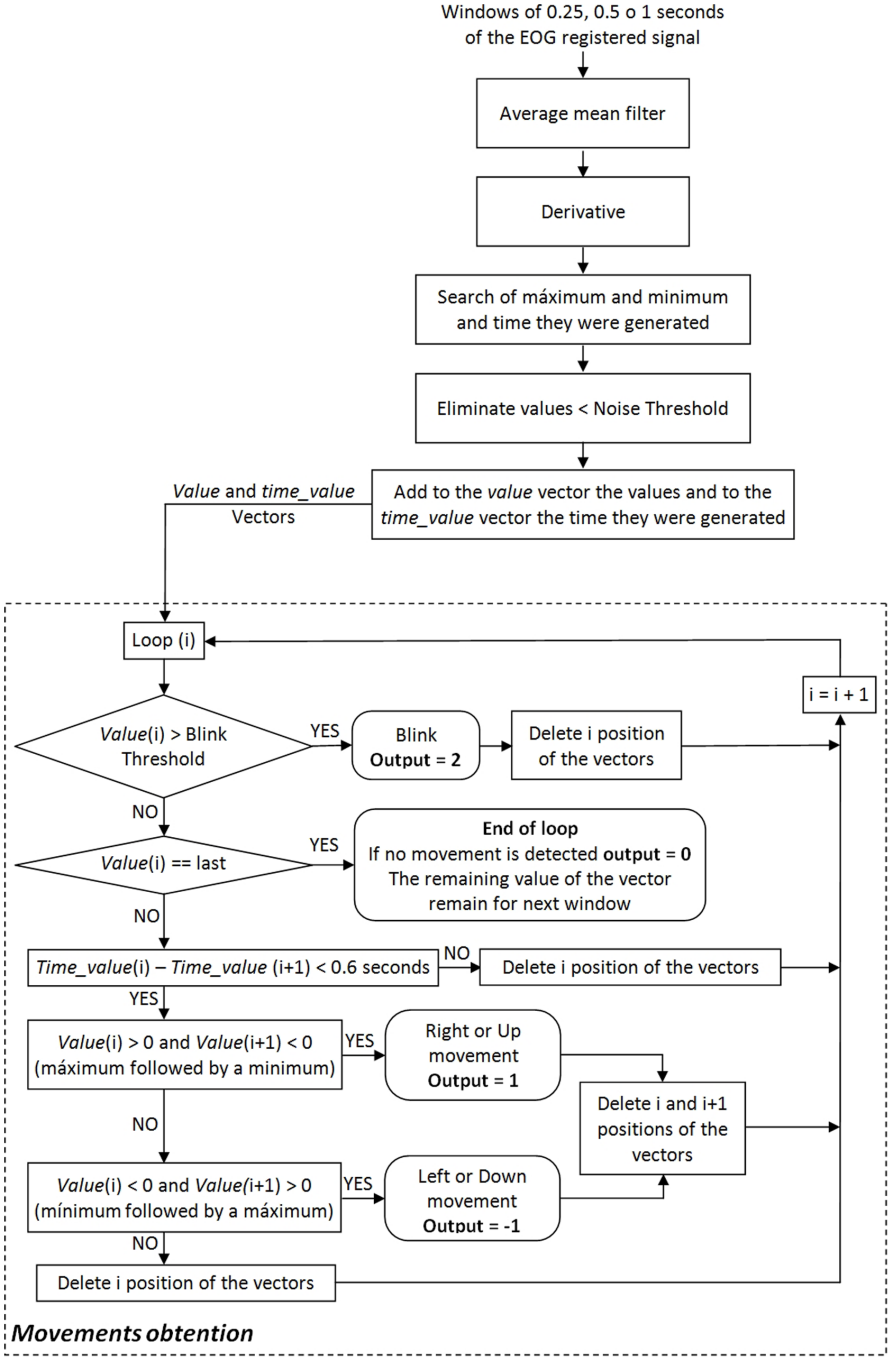
Processing algorithm. Procedure applied to the EOG signal to obtain the ocular movement in the current window. The output can be, 0 for no movement, 1 for a right/up movement, −1 for a left/down movement and 2 for a blink.

The algorithm has been designed registering the EOG signals with a frequency of 120 Hz. Figure 5 shows the algorithm diagram. First, the size of the EOG processing window is selected: 0.25 s, 0.5 s or 1 s. The algorithm is composed of the following steps:

1. A moving average filter is applied to get a clearer signal.
2. Next, the derivative is performed to detect abrupt changes of the signal when the user performs a fast movement of their eyes.
3. The maximums and minimums of the window are searched as well as when they are produced.
4. The values lower than the noise threshold are removed.
5. The rest of maximums and minimums values are added to a vector, including the time when they are produced.
6. Finally, this vector is evaluated in order to obtain the gaze direction and the blink:

- To detect a blink, it is checked if the value of the vector is higher than the blink threshold. In this case, the output of the algorithm will be blink (output  = 2), the value will be removed from the vector and the next iteration of the loop will start.
- In the last iteration of the loop, if the value does not correspond to a blink, the loop will end save this value for the next iteration of the algorithm, i.e. the next processing window. Thus, eye movements carried out between processing windows can be detected.
- Next, if a sequence of two values of the vector (maximum-minimum or minimum-maximum) is produced with a difference lower than 0.6 seconds, a right/up (output  = 1) or left/down (output = −1) movement has been produced. In this case, the detected sequence will be provided as output, both values will be removed from the vector and the next iteration of the loop will start. Otherwise, the first value of the sequence will be removed due to the fact that it is an isolated maximum or minimum that does not correspond to any movement, and the next iteration of the loop will start.
- If the value of the vector does not fulfill any previous condition, it will be removed from the vector.

This algorithm is applied independently to each channel (i.e. horizontal and vertical channel) in order to later combine the outputs provided by each one. This way, it is possible to detect right, left, up and down movements, and diagonal movements if both channels are combined. As it has been previously indicated, two thresholds (noise and blink thresholds) must be selected. These thresholds will be selected for each user and for each channel during the training, which must be performed before using the system.

The output of the algorithm will be 0 if any movement is detected, −1 if a left or down movement is detected, 1 if a right or up movement is detected, and 2 if a blink is detected.

If a processing window of 1 second was selected, the user would be able to perform two eye movements inside the processing window since an eye movement lasts about 0.5 s and the blink lasts less than 0.5 s. In this case, the algorithm would detect both movements and the output will be a vector with the values detected. Thus, this algorithm is more reliable and robust than our previous algorithm [10].

### Experimental Tests

In this section, the experimental tests performed to validate the EOG-based interface are explained. First, the graphical interfaces designed to perform the different tests are detailed. Finally, the test protocol followed by each user is described. In these tests, the EOG signals have been processed in windows of 0.25 seconds. Furthermore, diagonal movements have not been allowed in order to simplify the tests.

Two different experiments have been carried out. First, the success, no detection and error percentages have been obtained while the user performs specific ocular movements. Then, the users have performed several trajectories controlling a dot in the computer screen in order to reach several random targets. Ten volunteers, all healthy, with ages between 26 and 33 years old, carried out the experimental tests. User 1 had previous experience with this kind of interfaces. Human data presented in this article have been acquired under an experimental protocol approved by the ethics committee in the experimental research department of the Miguel Hernandez University of Elche, Spain. Written consent was obtained from each subject.

#### Graphical interfaces

**Figure 6:**
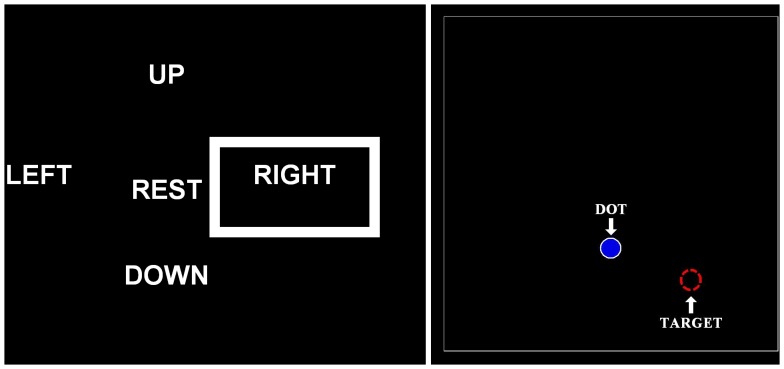
Graphical interfaces developed. Left: Graphical interface used for the training of the user and for obtaining the success, no detection and error percentages of the ocular algorithm. Right: Graphical interface used to reach the targets from the ocular movements of the user.

**Figure 7:**
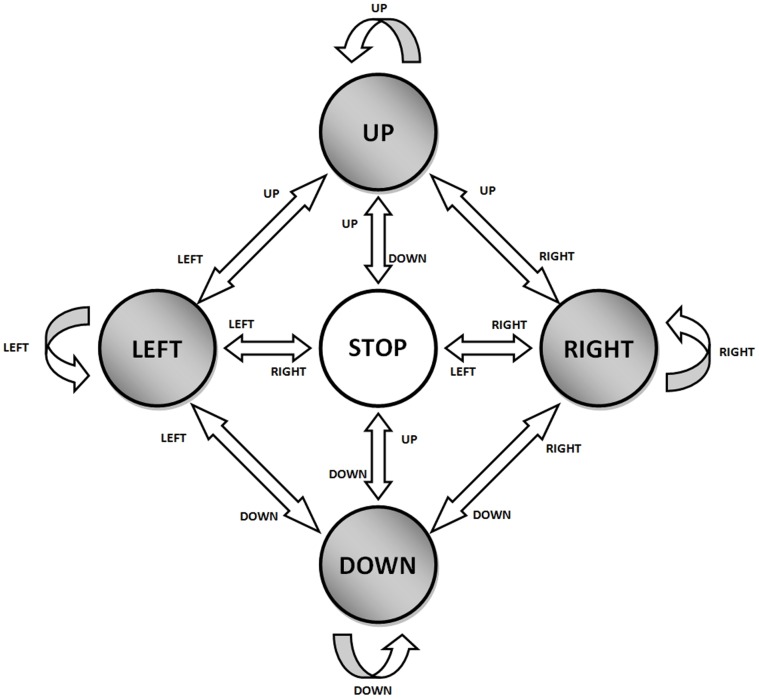
States machine used in the experimental tests. It allows performing change directions without going through the STOP state.

Three graphical interfaces to carry out the experimental tests as well as the training of the users have been designed in Matlab. The interfaces are described next:

1. **Graphical interface 1:** This interface, Fig. 6 (left), shows four directions (UP, DOWN, LEFT and RIGHT) and the rest state (REST). By default, REST is selected using a frame. When the user performs some ocular movement in one of the directions, the frame changes to this direction. If the user performs a blink, the active frame changes its colour a few seconds. This interface is used for training.
  - The movements between frames follow the states machine of Fig. 7. This states machine allows selecting different directions without passing through the rest state. For example, starting from the rest state, if the user performed an ocular movement to the right, the right state would be selected. Afterwards, if the user carried out an up ocular movement, the up state would be selected.
2. **Graphical interface 2:** The appearance of this interface is similar to the first one (Fig. 6, left), but it works in a different way. In this case, starting from the rest state, the direction where the user has looked at is selected by a frame during an instant, and then, the rest state is selected again. If the user performs a blink, the rest frame changes its colour during an instant, and then, it goes back to its original color. This interface is used to obtain the suitable thresholds of the EOG processing algorithm for each user and for training.
3. **Graphical interface 3:** The last interface (Fig. 6, right) has been designed so that the user can reach some targets. The user controls a dot that appears on the centre of the screen using their ocular movements.

- The workspace has a dimension of 840×840 pixels and the dot is moved 10 pixels each time a decision is taken. As four decisions are taken each second, the dot moves with a velocity of 40 pixels/second.
- The movement of the dot follows the states machine previously explained (Fig. 7). If the user performs an ocular movement to the right, the dot starts moving to the right; if then, the user performs an up ocular movement, the dot changes its movement direction to up. To stop the dot, an ocular movement in the opposite direction of the current movement must be done. In case the user blinks, the dot changes its colour if it has not been moved.
- In the experimental tests, the user must move the dot to a target that appears randomly on the screen by performing the appropriate ocular movements. The dot must be stopped as near as possible to the target and the user must carry out a blink to mark it.

#### Tests protocol

The users follow the next protocol:

1. The users are asked to perform several predefined ocular movements, which consist of looking right, left, up and down, and blink, using the graphical interface 2. According to the registered signals, suitable thresholds are selected for each user.
2. Afterwards, the users train freely with the system using the graphical interfaces 1 and 2. The second interface allow checking the selected thresholds. Not only does the graphical interface 1 allow testing the states machine previously explained, but it also allows the users to learn to control the movement on the screen. The selection of thresholds and the training lasts less than 5 minutes.
3. After the short training, the graphical interface 2 is used to obtain the success, no detection and error percentages of the EOG processing algorithm. To that end, a sequence of ocular movements are indicated to the user and it is checked if they are correctly detected, they are not detected or a wrong movement is detected. The sequence consists in indicating each movement five times to the user (right, left, up, down and blink). This sequence is repeated four times alternating the different movements. At the end, each movement is repeated 20 times. The users have a visual feedback of the ocular movements performed, so they know if they are doing well or not. Thus, they can continue their training in order to improve the ocular movements if it is necessary.
4. Finally, graphical interface 3 is used to perform 10 trajectories to reach several targets. The dot that the user must control appears in the centre of the screen. A target (a circumference) appears on the screen in a random position inside the screen. The user must carry out the necessary ocular movements to move the dot to the target and, after stopping it nearest the target, perform a blink to mark the target. The velocity index in relation to the minimum time required to reach the target has been measured as well as the position error produced to mark it.

## Results and Discussion

**Figure 8:**
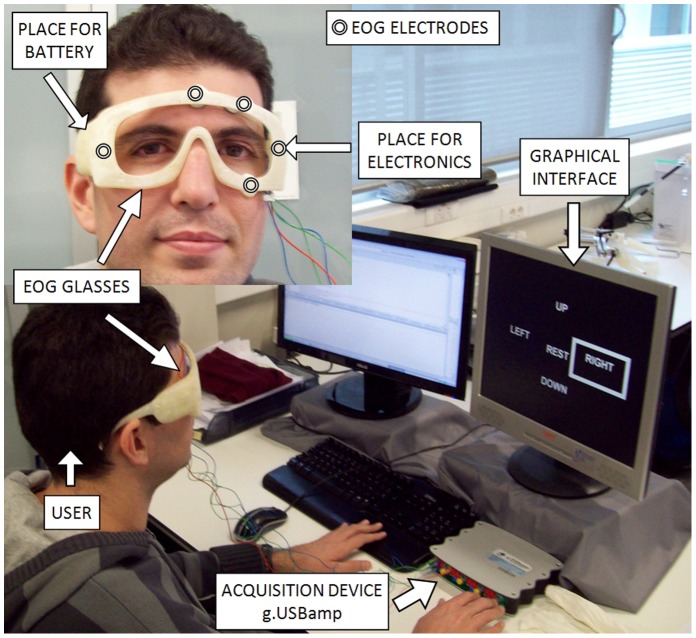
Local environment where the users perform the experimental tests. The subject of the photograph has given written informed consent, as outlined in the PLOS consent form, to publication of their photograph.

Several experiments have been made in order to validate the EOG-based interface. Once the suitable thresholds for each user have been selected and, after a short training, the reliability of the interface has been evaluated. Fig. 8 shows an image of the environment where the users perform the experimental tests.

Table 1 shows the results obtained with the second graphical interface. The ocular movement the users must carry out is indicated to them and it is checked if it is successfully detected, it is not detected or an error is produced. As it can be seen, the success percentages are very high with an average of 90%. 7% corresponds to no detections and the remaining, with an average of 3%, corresponds to error. All the errors are produced with the up ocular movements and the blink. This is because the EOG signals are very similar if an up ocular movement is performed strongly or a blink is done weakly. However, as it will be shown in the next tests, the users are able to reach the targets with low error and with high velocity index.

**Table 1:** Results of the validation of the EOG-based interface with the Average ± Standard Deviation (SD).

User	Success	No detection	Error
	%	%	%
1	90	8	2
2	86	5	9
3	94	5	1
4	96	4	0
5	85	14	1
6	89	9	2
7	79	16	5
8	85	10	5
9	98	1	1
10	95	1	4
Average±SD	90±6	7±5	3±3

Next, each user performs 10 repetitions with the third graphical interface. The user must carry out the necessary ocular movements to move the dot from the centre of the screen to the target, stop it, and perform a blink. In these tests, two parameters have been measured: the error and the velocity index.

- To measure the velocity index, the minimum time to reach and mark the target is calculated as well as the time actually used by the user. The velocity index is obtained dividing the minimum time by the required time by the user, i.e., how fast the user has been able to reach the target regarding the ideal minimum time. The minimum time is the time used to perform the shorter trajectory, i.e. doing the minimum number of movements to reach the target.
- On the other hand, the error has been measured using the Manhattan distance. This distance has been employed instead of the Euclidean distance because diagonal movements have not been taken into account to simplify the tests. The Manhattan distance, for two dots in a n-dimensional space (the target and the dot the user marks with the blink), is defined as:

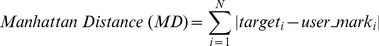
(1)If the formula is simplified for two dots in a bidimensional space:
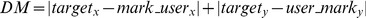
(2)


Table 2 shows the results obtained. As it can be seen, the users get a high velocity index with an average of 83%. The error obtained is also low (0.9). An error of 1.0 implicates that the user has marked a contiguous position to the target, i.e., there is a difference of 10 pixels (compared to the 840 pixels of the workspace). The dot moves with a velocity of 40 pixels/second. Therefore, it is difficult to carry out a finer control. The users have indicated that, during the tests, they are aware if they have performed an ocular movement wrongly, causing a no detection or an error. Thus, they can immediately correct the ocular movement or repeat the no detected movement.

**Table 2:** Results of the trajectories performed with the Average ± Standard Deviation (SD).

	Velocity index	Error
User	(%)	(Manhattan distance)
1	75	0.5
2	71	1.0
3	87	0.8
4	86	0.9
5	89	0.8
6	90	0.8
7	70	1.3
8	87	0.9
9	92	1.0
10	83	0.8
Average±SD	83±8	0.9±0.2

Finally, from the obtained results (time, error and distance to the target), a throughput index has been calculated. This throughput is based on the paper of X. Zhang and I.S. MacKenzie, where it is obtained based on the international standard ISO 9241-9 [20]. The throughput shown in (3) is calculated based on the time to reach the target (MT, in seconds), the standard deviation of the error position when marking the target (SD, in pixels) and the distance to the target (D, in pixels).
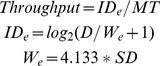
(3)


**Figure 9:**
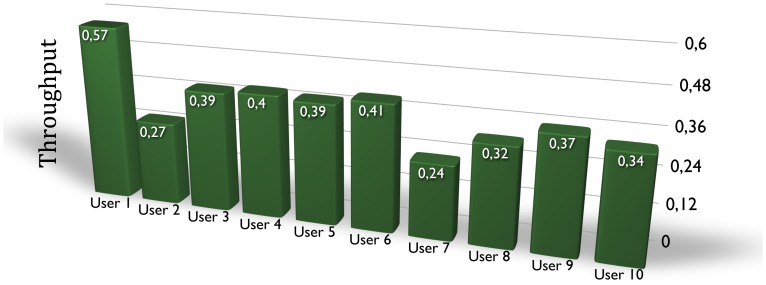
Throughput results.

Figure 9 shows the throughput results obtained for the five users. Users 3, 4 and 5 have similar indices according to the similar results obtained for their index velocity and distance errors. User 1, who has the smallest distance error, even though the index velocity is not so high, obtains the best throughput index. On the other hand, user 2 obtains the worst result due to getting the biggest distance error. The average throughput of the ten users is 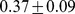.

Similar tests were performed in a previous work [21], where EOG technology was used to perform a velocity control of a graphical interface and a robot arm. The placement of the EOG electrodes was manual and they required conductive gel to work properly. The control velocity was achieved by obtaining the angle of the eye position through different EOG processing algorithm. Throughput was also calculated for three users, obtaining an average throughput of 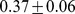 (User 1∶0.44, User 2∶0.35, User 3∶0.32). As it can be concluded from the results, the throughput is similar for both studies. Both studies allow the movement in a 2D plane and they have a velocity limitation. In the previous work, diagonals were allowed so as and the angle of the eyes determinate the velocity of the dot in the graphical interface, or the velocity of the end effector of the robot arm. In the present work, diagonals were not allowed to make the control simpler. Despite the differences between both works, the obtained throughput is similar. This implies that the interface designed in this work is more suitable for using due to its simplicity in controlling. Moreover, this interface allows placing dry electrodes in a simple way by using the pair of EOG glasses.

### Conclusion

In this work, a EOG-based human-computer interface that allows interacting with a computer has been described. A new pair of EOG glasses have been designed to improve the user's comfortability and to avoid the manual procedure of placing the EOG electrodes. Several tests have been made to validate the EOG interface, obtaining high accuracy, high velocity indexes and low error. The throughput index obtained is similar to other interfaces, even though the interface developed has a simpler control. Moreover, the pair of EOG glasses allow placing the dry electrodes in a simple way.

In future works, the electronic required to avoid the use of the current EOG amplifier will be integrated in the pair of EOG glasses in order to to develop an autonomous device. Also, several experiments will be performed with the pair of EOG glasses to test its comfortability.
